# Supplementary material for: Hydraulic transmissivity inferred from ice-sheet relaxation following Greenland supraglacial lake drainages
Source: Nat Commun. 2021 Jun 25;12:3955. doi: 10.1038/s41467-021-24186-6 (PMC8233380; doi:10.1038/s41467-021-24186-6)
Supplement: Supplementary file 1 — SUPPLEMENTARY INFO [file 41467_2021_24186_MOESM1_ESM.pdf]

Supplementary information for
Hydraulic transmissivity inferred from ice-sheet relaxation following Greenland supraglacial lake drainages

Ching-Yao Lai*, Laura A. Stevens, Danielle L. Chase, Timothy T. Creyts,
Mark D. Behn, Sarah B. Das, Howard A. Stone

*Correspondence to: cylai@princeton.edu

The following sections include:

Supplementary Figures 1 to 8
Supplementary Tables 1 to 3

1 Blister relaxation model

We consider the elastic relaxation of a fluid-filled blister (cavity) beneath an infinite elastic medium and above a porous substrate. During the relaxation the fluid in the blister leaks into the porous substrate below and ahead of the blister. The radius of the blister R remains unchanged (experimental observation) and the total volume V_{tot} of fluid in both the blister $V(t)$ and the porous layer is fixed.

1.1 Mass balance

The radial and vertical velocities in the blister are denoted u and w, respectively. The continuity equation for incompressible flow $\nabla \cdot \mathbf{u} = 0$ in cylindrical coordinates, assuming axisymmetry, is

$$\frac{1}{r}\frac{\partial}{\partial r}(ru) + \frac{\partial w}{\partial z} = 0. \tag{S.1}$$

Integrating the equation vertically from the bottom $z = 0$ to the top $z = h(r,t)$ of the blister

$$\int_0^h \frac{1}{r}\frac{\partial}{\partial r}(ru)dz + w|_{z=h} - w|_{z=0} = 0. \tag{S.2}$$

Using Leibniz's rule, equation (S.2) becomes

$$\frac{1}{r}\frac{\partial}{\partial r}\int_0^h (ru)dz - u|_{z=h}\frac{\partial h}{\partial r} + w|_{z=h} - w|_{z=0} = 0. \tag{S.3}$$

At $z = h(r,t)$, $\mathrm{D}/\mathrm{D}t(z - h(r,t)) = 0$ where $\mathrm{D}/\mathrm{D}t \equiv \partial/\partial t + \mathbf{u}\cdot\nabla$ is the material derivative. Thus $-\partial h/\partial t - u\partial h/\partial r + w = 0$ at $z = h(r,t)$ and equation (S.3) becomes

$$\frac{1}{r}\frac{\partial}{\partial r} r\int_0^h udz + \frac{\partial h}{\partial t} - w|_{z=0} = 0. \tag{S.4}$$

Define the average velocity in the blister as $\bar{u} \equiv h^{-1}\int_0^h udz$ so that equation (S.4) becomes

$$\frac{1}{r}\frac{\partial}{\partial r}(r\bar{u}h) + \frac{\partial h}{\partial t} - w_0 = 0, \quad \text{(blister)}, \tag{S.5}$$

which governs mass conservation in the blister.

In the porous substrate of constant thickness h_0 and porosity ϕ, the layer thickness is constant $\partial h_0/\partial t = 0$. The flow velocity in the porous layer can be describe by Darcy's law with velocity $u_p(r,t)$. Following the same steps as equations (S.1-S.5) and substituting w_0 for the vertical velocity at the interface between the blister and porous layer, we obtain

$$\frac{1}{r}\frac{\partial}{\partial r}(r\phi u_p h_0) + w_0 = 0, \quad \text{when} \quad r < R \quad \text{(porous layer)}. \tag{S.6}$$

Equations (S.5) and (S.6) give

$$\frac{\partial h}{\partial t} + \frac{1}{r}\frac{\partial}{\partial r}(r\bar{u}h) + \frac{1}{r}\frac{\partial}{\partial r}(r\phi u_p h_0) = 0, \quad \text{when} \quad r < R. \tag{S.7}$$

The height change of the blister is balanced by the radial gradient of fluid flux not only in the blister but also the porous layer. Assuming axisymmetry, it is convenient to integrate the blister mass conservation equation (S.5) radially

$$\int_0^R \frac{\partial}{\partial r}(r\bar{u}h)dr + \int_0^R r\frac{\partial h}{\partial t}dr - \int_0^R rw_0 dr = 0 \tag{S.8}$$

Since the volume of the blister is $V(t) = 2\pi\int_0^R rhdr$, and no fluid is injected during relaxation so that $r\bar{u}h|_0 = 0$, the above equation gives the global mass conservation law for the blister

$$\frac{\mathrm{dV}}{\mathrm{dt}} - \int_0^R 2\pi r w_0 dr = 0, \tag{S.9}$$

where the first term is the rate of blister volume reduction and the second term is the total flux of liquid leaking from the blister into the porous substrate ahead of the blister. Similarly, integrating the mass balance equation (S.6) in the porous layer radially and applying $ru_p h_0|_0 = 0$ (no fluid is injected during relaxation), we obtain

$$r\phi u_p h_0|_R + \int_0^R rw_0 dr = 0. \tag{S.10}$$

Ahead of the blister ($r > R$), $w_0 = 0$ since there is no vertical fluid flux. Thus $\partial(r\phi u_p h_0)/\partial r = 0$, i.e., the horizontal flux $r\phi u_p h_0$ is constant along r. Thus

$$r\phi u_p h_0|_r = R\phi u_p h_0|_R. \tag{S.11}$$

Combining equations (S.9, S.10, S.11), we obtain the global mass conservation in the porous layer for $r > R$

$$\frac{\mathrm{dV}}{\mathrm{dt}} = -2\pi r\phi u_p h_0, \quad \text{when} \quad r > R \quad \text{(porous layer)}. \tag{S.12}$$

1.2 Force Balance

During the formation of the water-filled blister at the ice-bed interface, the deformation of ice imposes elastic stresses on the blister. As water in the blister leaks into the porous substrate, the elastic stresses relax. While the elastic stresses act as a driving force for the blister relaxation, the viscous stresses in the fluid flow (along the r direction) resist the relaxation. We now give an approximate description of the time-dependent relaxation. The pressure source in the system, i.e. the elastic driving stresses $P(t)$, is reduced along the the flow due to the radial pressure gradient $\partial p/\partial r < 0$ that drives the viscous flow in the porous substrate, and eventually reaches the background pressure. The background pressure (the overburden pressure of ice) is assumed constant with space and time around the blister, thus does not affect the relaxation dynamics (see Supplementary Information Section 2). Assuming the viscous resistance in the blister is negligibly small compared with the viscous resistance in the porous layer (see Supplementary Information Section 1.5), the time-dependent dynamics can be thus modeled as

$$P(t) + \int_R^{R_p(t)} \frac{\partial p(r,t)}{\partial r} dr = 0, \tag{S.13}$$

where $R_p(t)$ is the position of the advancing front of the liquid in the porous substrate. For convenience, we introduce a characteristic height scale $H(t) = h(r = 0, t)$ and use the fixed blister radius R as the radial length scale so that

$$r = Rs, \quad \text{and} \quad h(r,t) = H(t)\Omega(s), \tag{S.14}$$

where s and $\Omega(s)$ are dimensionless. Note that here we assume the dimensionless cavity shape $\Omega(s)$ is independent of time, i.e., the relaxation dynamics is self-similar [1]. The total volume of the blister can be written as

$$V(t) = 2\pi \int_0^R rh(r,t)dr = 2\pi H(t)R^2\alpha, \quad \text{where} \quad \alpha \equiv \int_0^1 s\Omega(s)ds \tag{S.15}$$

is a time-independent dimensionless parameter. The exact elastic stress distribution $p(r,t)$ around the blister is a function of the shape of the cavity $h(r,t)$ (equation (2.1) in [2]). To compare the model with experimental and field data, we will derive the solution for the blister volume $V(t)$ as a function of time.

For small elastic strains, $H/R \ll 1$, the elastic stresses, analogous to Hooke's law, scale linearly with the vertical displacement $P(t) \propto H(t)$. Here, using equation (2.1) in [2] and (S.15), we approximate the elastic stress with the time-dependent pressure

$$P(t) = \frac{E}{2(1-\nu^2)} \frac{H(t)}{R} = \frac{E}{2(1-\nu^2)} \frac{V(t)}{2\alpha\pi R^3}. \tag{S.16}$$

1.2.1 Porous substrate

Fluid flow through a porous medium obeys Darcy's law, where the (radial) fluid flux q (volume per unit time per unit area crossing the flow) is linearly proportional to the pressure gradient $\partial p/\partial r$. In glaciology the same approach was used by Weertman to describe the water flow through a porous rock with a uniform permeable layer thickness below the ice sheet [3]. Note that the fluid flux q is related to the fluid velocity u_p in a porous medium with porosity ϕ (volume of pores normalized by total volume) via $q = \phi u_p$, since fluid flux is only contributed by the flow in the pores. For a fluid with viscosity μ and permeability k, Darcy's law gives

$$\frac{\partial p}{\partial r} = -\frac{\mu}{k} q = -\frac{\mu\phi}{k} u_p. \quad \text{(porous layer)} \tag{S.17}$$

Integrating equation (S.17) and using equation (S.12), we obtain

$$\int_R^{R_p(t)} \frac{\partial p}{\partial r} dr = \int_R^{R_p} \frac{-\mu\phi u_p}{k} dr = \frac{\mu}{2\pi h_0 k} \frac{dV}{dt} \ln\left(\frac{R_p}{R}\right). \tag{S.18}$$

Since the total water volume V_{tot} is a combination of water in the blister and the porous substrate, $V_{tot} = V(t) + \phi\pi h_0 R_p(t)^2$. Thus

$$\frac{R_p(t)}{R} = \sqrt{\frac{V_{tot} - V(t)}{\phi\pi h_0 R^2}}. \tag{S.19}$$

Equations (S.18) and (S.19) give the pressure drop along the flow in the porous layer as a function of $V(t)$

$$\int_R^{R_p(t)} \frac{\partial p}{\partial r} dr = \frac{\mu}{4\pi h_0 k} \frac{dV}{dt} \ln\left(\frac{V_{tot} - V(t)}{\phi\pi h_0 R^2}\right), \quad \text{for} \quad r > R \tag{S.20}$$

Finally, substituting equations (S.16) and (S.20) into (S.13), we obtain a first-order ordinary differential equation that governs the time evolution of the blister volume $V(t)$,

$$\frac{E}{2(1-\nu^2)} \frac{V}{2\alpha\pi R^3} + \frac{\mu}{4\pi h_0 k} \ln\left(\frac{V_{tot} - V}{\phi\pi h_0 R^2}\right) \frac{dV}{dt} = 0, \tag{S.21}$$

with an initial blister volume $V(t = 0) = V_i$. The second term in the above equation result from the viscous dissipation in the porous substrate, respectively. The first term represents the driving force of the system, the elastic stresses.

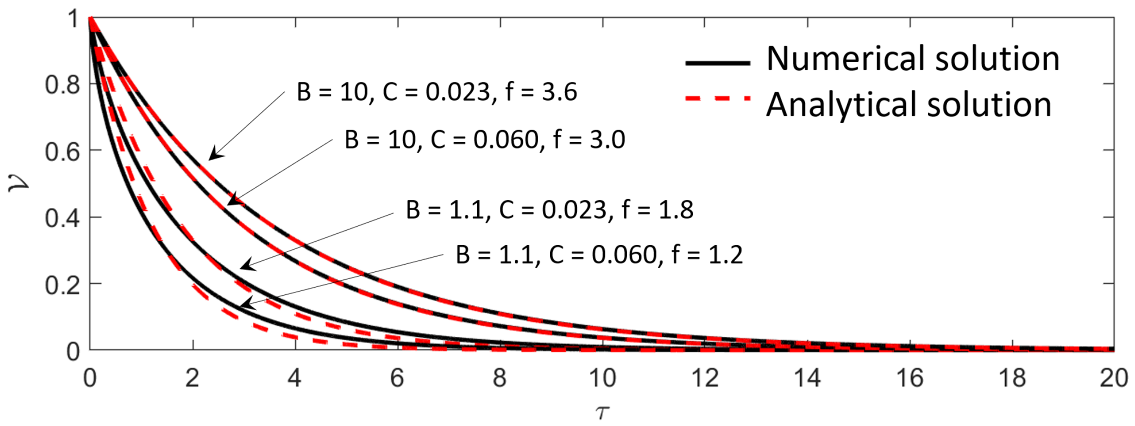

Supplementary Figure 1: Model solutions. The dimensionless blister volume $\mathcal{V}$ as a function of dimensionless time τ, defined in equation (S.22). The analytical exponential solutions (equation (S.27), red dashed curves) approximate the numerical solutions (black solid curves) of the full non-linear ODE (equation (S.23)) for a range of B, C (defined in equation (S.24)) and the corresponding f (defined in equation (S.26)).

1.3 Non-dimensionalization

For convenience, we define the dimensionless volume and time as

$$\mathcal{V} \equiv \frac{V}{V_i} \quad \text{with} \quad \tau \equiv t\frac{Ekh_0}{\mu(1-\nu^2)R^3}, \tag{S.22}$$

so that equation (S.21) can be non-dimensionalized

$$\mathcal{V} + \alpha \ln\left(\frac{B-\mathcal{V}}{C}\right)\frac{d\mathcal{V}}{d\tau} = 0, \quad \text{I.C.} \quad \mathcal{V}(0) = 1, \tag{S.23}$$

$$\text{where} \quad B \equiv \frac{V_{tot}}{V_i} \quad \text{and} \quad C \equiv \frac{\phi\pi h_0 R^2}{V_i}. \tag{S.24}$$

Although $V(t)$ depends on nine parameters $E, \nu, \mu, R, h_0, k, \phi, V_i, V_{tot}$ (equation (S.21)), we found that the behaviour of the dimensionless volume $\mathcal{V}$ as a function of dimensionless time τ depends on two dimensionless parameters B and C. B is the ratio between total water volume injected into the system and initial blister volume and C is the ratio between water volume just beneath the blister and the initial blister volume. Based on field data $B \approx 1.14$ (based on the initial blister volume V_0 and lake volume V_{tot} estimated for the 2012 North lake drainage event [4]), and $C = 0.04 - 0.11$ (Table 1). The numerical solutions to equation (S.23) for a range of B, C are plotted in Supplementary Fig. 1 as black curves.

1.4 Analytical solution

Since $\ln\left(\frac{B-\mathcal{V}}{C}\right)$ varies slowly with $\mathcal{V}$, it can be approximated with a time-independent constant $\ln\left(\frac{B-\gamma}{C}\right)$, where γ is a numerical constant to be determined below. Equation (S.23) can be approximated with a linear ODE

$$\mathcal{V} + f\frac{d\mathcal{V}}{d\tau} = 0, \quad \text{with} \quad \mathcal{V}(0) = 1, \tag{S.25}$$

$$\text{where} \quad f \equiv \alpha \ln\left(\frac{B-\gamma}{C}\right) \tag{S.26}$$

and yields an exponential solution for $\mathcal{V}$

$$\mathcal{V} = \exp\left(-\frac{\tau}{f}\right). \tag{S.27}$$

To find the value of γ so that equation (S.25) approaches (S.23), we choose γ to be $\mathcal{V}$ averaged over a time scale of f, i.e., $\gamma = f^{-1}\int_0^f \mathcal{V}d\tau = \int_0^f \exp(-\tau/f)d\tau = (e-1)/e \approx 0.63$. Supplementary Fig. 1 shows that the exponential solution (red dashed curve, equation (S.27)) is a good approximation to the numerical solution (black solid lines) to the non-linear ODE (equation (S.23)).

To compare with the field data, we also obtain a solution for the blister height $h(r,t)$. The height of the blister at any location directly relates to the dimensionless volume. Since the blister radius remains unchanged during relaxation, the volume change results from a change in height. The blister height $h(r,t)$ relates to its initial value $h(r, t=0)$ via the formula

$$\mathcal{V} = \frac{V}{V_i} = \frac{2\pi\int_0^R h(r,t)rdr}{2\pi\int_0^R h(r,t=0)rdr} = \frac{2\pi\alpha'(r)R^2h(r,t)}{2\pi\alpha'(r)R^2h(r,t=0)} = \frac{h(r,t)}{h(r,t=0)} = \mathcal{H}, \tag{S.28}$$

where $\alpha'(r) \equiv \alpha H(t)/h(r,t)$ is related to the self-similar shape of the blister and so is independent of time and space. Therefore the height of the blister, measured at a given location, rescaled by its initial value $\mathcal{H}$, also has an exponential response

$$\mathcal{H} = \exp\left(-\frac{\tau}{f}\right), \tag{S.29}$$

which is directly compared with field data in Fig. 3 in the main text. The numerical pre-factor f can be calculated for each field data set using B, C, and $\alpha \approx 0.32$ (found experimentally from fitting the experimental data to equation (S.27)). We estimate that for the nine observational data sets presented in Fig. 3 in the main text $f \approx 0.5 - 0.8 = \mathcal{O}(1)$ (Table 1), which is remarkably close to the experimental value $f \approx 0.6 - 0.7$. The effect of such range of f on the solution (equation (S.27)) is shown in Supplementary Fig. 1.

All relevant parameters used in this paper (dimensionless and dimensional) for both the experimental system and field observations are listed in Table 1.

1.5 Viscous dissipation in the blister

In this section we show the negligible viscous dissipation in the blister compared with that in the porous sheet. Below We calculate the viscous pressure drop along the flow in the blister $\Delta p_{vb} = \int_0^R \partial p/\partial r dr$ and compare it with the viscous pressure drop in the porous sheet $\Delta p_{vp} = \int_R^{R_p} \partial p/\partial r dr$ (equation (S.20)).

In the limit where viscous effects are important relative to inertial effects, i.e. a representative Reynolds number $Re_{\text{eff}} = \rho \bar{u} H^2/(\mu R) \ll 1$, the flow in the blister is laminar. We solved the Stokes equation $\partial p/\partial r = \mu \partial^2 u/\partial z^2$ (neglecting the r-derivatives terms since $z \ll r$) with boundary conditions $u(z = h) = 0$ and $u(z = 0) = \phi u_p$, and obtain a variant of Darcy's law,

$$\frac{\partial p}{\partial r} = -\frac{\mu}{h^2 S_\ell}\bar{u}, \quad \text{where} \quad S_\ell \equiv \left(\frac{1}{12} + \frac{k}{2h^2}\right), \quad \text{(blister)} \tag{S.30}$$

where S_ℓ is a slip factor that couples the effect of non-zero velocity at the interface between the blister and porous layer. Equations (S.7), (S.17), and (S.30) give

$$\frac{\partial h}{\partial t} - \frac{1}{r}\frac{\partial}{\partial r}\left(\frac{r}{\mu}(h^3 S_\ell + h_0 k)\frac{\partial p}{\partial r}\right) = 0, \quad \text{when} \quad r < R \tag{S.31}$$

Rearranging equation (S.31), we obtain

$$\frac{\partial p}{\partial r} = \frac{\frac{\mu}{r}\int_0^r \frac{\partial h}{\partial t} r' dr'}{h^3 S_\ell + h_0 k} \tag{S.32}$$

The integrated pressure drop along the flow in the blister, considering the velocity slipping against the porous layer, is thus

$$\int_0^R \frac{\partial p}{\partial r} dr = \int_0^R \frac{\frac{\mu}{r}\int_0^r r' \frac{\partial h}{\partial t} dr'}{h^3/12 + hk/2 + h_0 k} dr, \quad \text{for} \quad r < R \tag{S.33}$$

To rewrite the pressure drop in terms of $V(t)$, we substitute equations (S.14) and (S.15) into (S.33) to find

$$\int_0^R \frac{\partial p}{\partial r} dr = \frac{\mu R^2}{H^3}\frac{dH}{dt}\int_0^1 \frac{s^{-1}\int_0^s \Omega(s) s' ds'}{\Omega(s)^3/12 + \Omega(s)k/(2H^2) + h_0 k/H^3} ds = \frac{4\pi^2 \alpha^2 \mu R^6}{V^3}\frac{dV}{dt}\beta. \tag{S.34}$$

$$\text{where} \quad \beta \equiv \int_0^1 \frac{s^{-1}\int_0^s \Omega(s) s' ds'}{\Omega(s)^3/12 + \Omega(s)k/(2H^2) + h_0 k/H^3} ds \tag{S.35}$$

is a dimensionless parameter. Although $H(t)$ is time-dependent, the typical transmissivity $kh_0 \approx \mathcal{O}(1) - \mathcal{O}(10^2)$ mm^3 (Fig. 3 in the main text), porous layer thickness $h_0 \approx 0.1$ m, and blister height $H \approx 1$ m give $k/H^2 \ll 1$, $h_0 k/H^3 \ll 1$. Thus β can be approximated as $\beta \approx \int_0^1 \frac{s^{-1}\int_0^s \Omega(s) s' ds'}{\Omega(s)^3/12} ds$, which is independent of time.

Combining equations (S.20) and (S.34), the viscous dissipation in the blister compared with that in the porous sheet can be quantified by a dimensionless parameter

$$A \equiv \frac{\Delta p_{vb}}{\Delta p_{vp}} = \frac{\int_0^R \partial p/\partial r dr}{\int_R^{R_p} \partial p/\partial r dr} \approx \frac{16\pi^3 R^6 k h_0}{V_i^3}.$$

Based on field data $A = 5{\times}10^{-8}{-}2{\times}10^{-6} \ll 1$ (Table 1), thus the viscous resistance in the blister is negligible compared to that in the porous sheet. The dominant viscous resistance in the porous sheet ($A \ll 1$) yields a simple balance between the $\mathcal{V}$ and $d\mathcal{V}/d\tau$ terms in equation (S.25), resulting in an exponential solution (equation (S.27)). In our experiments, we design the parameters so that $A \approx 0.001 - 0.002 \ll 1$ (Table 1) and we find that the experiments agree with the exponential solution (Fig. 2d in the main text).

1.6 Pre-filled porous substrate

If water fully fills the subglacial drainage system prior to the blister relaxation, the water in the blister could enter the water-filled porous sheet by deforming the pores (Section 4.5 in Hewitt et al. (2018) [5]). Alternatively, if we assume that the water pressure in the drainage system at a radial distance R_o away from the blister center is unperturbed by the formation and relaxation of blister and within $r < R_0$ the drainage system is fully filled with water, the blister height still obeys $\mathcal{H} = \exp(-\frac{\tau}{f})$ (equation (S.29)) where the numerical pre-factor is defined as $f \equiv \alpha \ln(R_0/R)^2$. In reality R_0 could be determined by the location of partially empty channels and sheets [6] where the subglacial drainage system is "under-pressured" [6] due to the exposure of subglacial water pressure to atmospheric pressure; this is similar to the empty part of the porous substrate in our experiments. Note that when R_0 changes from 10 to 50 km f only increases from 1 to 2, which is of the same order of magnitude as the value calculated for the model with a partially filled porous sheet ($f \approx 0.5 - 0.8$ (Table 1)). Thus the inferred transmissivity kh_0 for a pre-filled drainage system is of the same order of magnitude as that for a partially filled porous sheet.

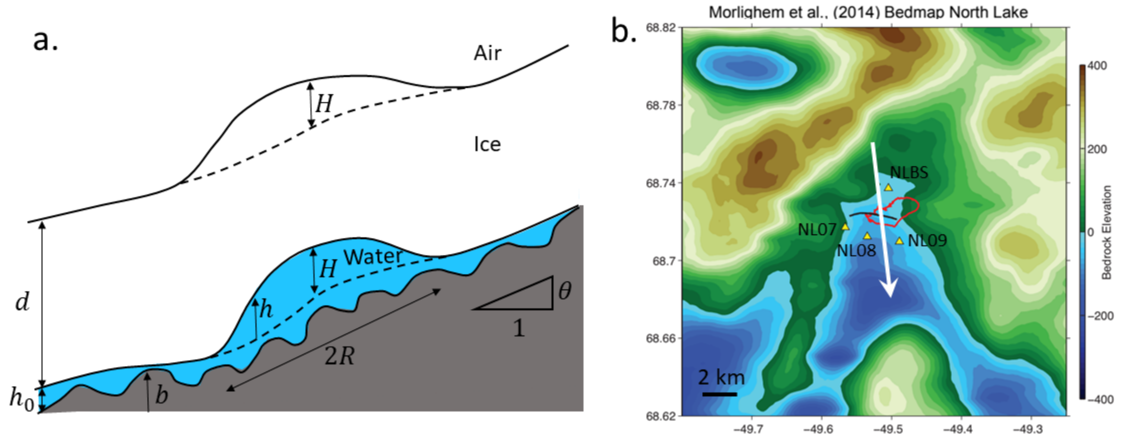

Supplementary Figure 2: **A blister on a inclined bed.** **(a)** Schematic of a blister of radius R and characteristic height H under an ice sheet of thickness d on a bed with spatially varying bed elevation b and characteristic slope θ. The characteristic thickness of the drainage system is h_0. Dashed lines denote the ice surface and bottom after the ice-sheet relaxation. **(b)** The bed topography near the North Lake inferred by Morlighem et al. (2014) [7]. The triangles, black curve, and red contour are the GPS stations, a vertical hydrofracture through which the lake drains, and the 2011 Lake contour, respectively. The largest bed slope, $\theta \approx 0.04$, aligns with the white arrow.

2 Effects of bed slope, ice overburden, and hydrostatic pressure on blister relaxation

The blister model in this paper assumes that (1) bed slope, (2) ice overburden pressure, and (3) hydrostatic pressure of water in the blister have negligible effects on the ice-sheet relaxation dynamics. In this section we estimate these effects. On an inclined bed with elevation b, the water flux $\mathbf{q}$ in the subglacial water sheet as a function of the hydraulic potential ϕ is

$$\mathbf{q} = -\frac{k}{\mu}\nabla\phi. \tag{S.37}$$

The hydraulic potential involves a bed elevation-related contribution $\rho_w g b$ and the water pressure p in the water sheet

$$\phi = \rho_w g b + p \quad \text{and} \quad p = \rho_i g d + \rho_w g h + \Delta p_e, \tag{S.38}$$

where ρ_i and ρ_w are the density of ice and water, respectively, d is the ice-sheet thickness, h is the blister height, and Δp_e is the the third term in the water pressure that balances the elastic stresses of the deformed ice sheet due to the blister. The horizontal hydraulic potential gradient in equation (S.37) can be written as

$$\nabla\phi = \rho_w g \nabla b + \rho_i g \nabla d + \rho_w g \nabla h + \nabla(\Delta p_e) \tag{S.39}$$

Below we estimate the magnitude of each term in equation (S.39) and find that the elastic-stress term dominates. For a blister (height $H \approx 1$ m and radius $R \approx 2$ km) under an ice sheet (Young's modulus $E \approx 10$ GPa), the gradient of elastic stresses is $\nabla(\Delta p_e) \approx EH/R^2 \approx 2500$ Pa/m. The bed slope beneath and around the blister is roughly $\theta \approx 0.04$. The bed-elevation related term in equation (S.39) is $\rho_w g \nabla b \approx \rho_w g \theta \approx 400$ Pa/m $\ll \nabla(\Delta p_e)$, which is small compared with the elastic term.

In addition, the ice overburden pressure is negligible due to the small variation of ice-sheet thickness d over the blister length scale $\rho_i g \nabla d \ll \nabla(\Delta p_e)$. The contribution of the hydrostatic pressure due to the water height in the blister is $\rho_w g \nabla h \approx \rho_w g H/R \approx 5$ Pa/m $\ll \nabla(\Delta p_e)$, and also is also negligibly small. Thus (1) bed elevation, along with (2) overburden pressure of ice, and (3) the hydrostatic pressure in the blister contribute little to the hydraulic potential gradient (equation (S.39)) when a blister is considered, and have small effects on the water flux q compared with the blister-induced elastic stress. For simplicity we neglect these effects in the blister relaxation model (Supplementary Information Section 1). Note that the bed slope needs to be as large as $\theta > EH/(R^2 \rho_w g) \approx 0.25$ to have a non-negligible effect on blister relaxation.

Therefore in this paper the water flux in the water sheet is approximately $\mathbf{q} = -k/\mu\nabla p$, where the water pressure in the blister is of the same order of magnitude as the elastic stress $p = \mathcal{O}(EH/R)$, which is the dominant driving stress for the water flow. Note that when the elastic stress has mostly relaxed and the blister thickness is much less than $H < \rho_w g \theta R^2/E \approx 16$ cm, the bed topography becomes the dominant mechanism to drive the subglacial flow.

3 Viscous effects of ice

The blister model assumes that ice is elastic. In this section we estimate the effect of viscous flow in ice sheets on the relaxation of a blister. We consider an extreme case where ice is purely viscous and estimate the surface elevation change h_{visc} compared with that resulting from a pure elastic ice h_{elast}.

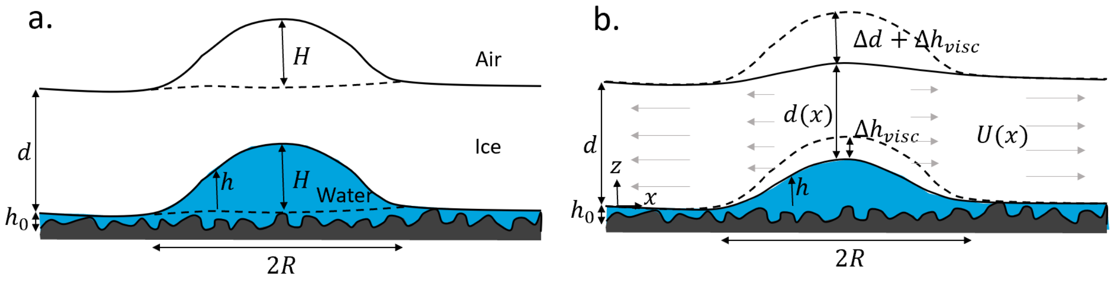

Supplementary Figure 3: **Blister under a purely viscous ice sheet. (a)** A water-filled blister. The dashed lines mark the relaxed positions of the top and bottom of the ice sheet. **(b)** The decrease of surface elevation between the beginning of blister relaxation (dashed line, same as the solid line in **(a)**) and a certain time Δt afterwards (solid line) is a combination of the thinning of blister height Δh_{visc} and the thinning of ice sheet thickness Δd by ice flowing (gray arrows) with varying velocity $U(x)$.

3.1 Viscous ice sheet

When a viscous ice layer is on top of a water-filled blister, the lowering of the vertical surface elevation could result from the (1) thinning of ice-sheet thickness Δd and the (2) thinning of the blister thickness Δh_{visc} (Supplementary Fig. 3). Below we estimate both contributions.

3.1.1 Ice-sheet thinning

When a viscous layer of ice is on top of a water-filled blister, the cross-sectional velocity is uniform U (Supplementary Fig. 3b) due to the free-slip top and bottom boundary conditions. Due to mass conservation the thinning of ice-sheet thickness depends on the horizontal gradient of vertical flux $\partial d/\partial t + \partial/\partial x(Ud) = 0$. Note that where ice sits on top of the drainage water sheet the shear stress at the ice-bed interface can be nonzero depending on the characteristics of the ice-bed contact. Here we only consider a case with zero shear stress at the bed, which overestimates the ice thinning Δd. The inclined ice-water interface $z = h$ and non-uniform ice thickness d contribute to the hydraulic potential gradient $\nabla(\rho_i gh + \rho_i gd)$ driving the ice flow. Note that initially $\nabla d \approx \nabla h$ so the total hydraulic potential gradient is on the same order of magnitude as $\rho_i g \nabla h$. Resistance in the ice flow comes from the horizontal gradient of the deviatoric stress τ_{xx}, where x is the along-flow direction. The balance of the driving and resisting mechanism gives $\tau_{xx}/R \approx \rho_i gH/R$. The rheology of ice is given by Glen's flow law [8] $\tau_{xx} \approx B\dot{\epsilon_{xx}}^{1/n} \approx B(U/R)^{1/n}$ where $n \approx 3$ and B is temperature-dependent. Thus $U \approx (\rho_i gH/B)^n d$. Along with mass conservation, we obtain the rate of ice thinning scales as

$$\frac{\Delta d}{\Delta t} \approx \frac{Ud}{R} \approx \left(\frac{\rho_i gH}{B}\right)^n d. \tag{S.40}$$

We find that for a typical range of the viscosity factor $B \approx 2.7 \times 10^5 \sim 10^6$ Pa·year$^{1/3}$ (at ice temperature $T = -30 \sim -5$°C) [9], $H \approx 1$ m and $d \approx 1$ km, so that after $\Delta t = 10$ days the ice only thins $\Delta d \approx 0.02 - 1$ mm.

3.1.2 Blister thinning

The driving force that causes the thinning of the blister Δh_{visc} (Supplementary Fig. 3b) is the gradient of hydrostatic pressure in the blister $\rho_w g \nabla h$, because there are now no elastic stresses in the ice sheet. The resisting mechanism for relaxation of the surface elevation is thus the viscous dissipation of water flux q in the water sheet of thickness h_0 and effective permeability k. A balance between the driving hydrostatic pressure and resisting viscous dissipation along the water flux is $\rho_w gH \approx \mu qR/k$. The rate of blister volume reduction supplies the water flux in the water sheet, i.e., $\Delta V/\Delta t \approx qh_0R$. Here we drop the $\mathcal{O}(1)$ pre-factors (e.g. π). The decrease in vertical blister height scales with a decrease in volume as $\Delta h_{visc}R^2 \approx \Delta V$. Therefore the rate of blister thinning under a viscous ice sheet scales as

$$\frac{\Delta h_{visc}}{\Delta t} \approx \frac{\rho_w gHkh_0}{R^2\mu}. \tag{S.41}$$

We find that for water viscosity $\mu \approx 1$ mPa·s, sheet transmissivity $kh_0 \approx 1$ mm^3, and the typical values of H, R used above, after $\Delta t = 10$ days the blister thins $\Delta h_{visc} \approx 2$ mm.

3.2 Elastic ice sheet

On the other hand, in the original blister model ice is considered elastic and the relaxation driving force is the elastic stress cause by the ice-sheet deformation. The driving elastic stress and resisting viscous dissipation along the water flux give $EH/R \approx \mu qR/k$. In the same way as for viscous ice, the volume conservation of water gives $\Delta V/\Delta t \approx qh_0R$ and $\Delta h_{elast}R^2 \approx \Delta V$. The rate of decrease in blister height due to the elastic relaxation of ice sheet is thus

$$\frac{\Delta h_{elast}}{\Delta t} \approx \frac{EHkh_0}{R^3\mu}. \tag{S.42}$$

3.3 Comparison

Comparing equations (S.40), (S.41), and (S.42), we find that within a given time period Δt and the typical parameter values of E, R, H, kh_0, d, μ, B used above, the vertical blister height change under a purely elastic ice sheet is much larger than the vertical surface elevation change under a purely viscous ice sheet.

$$\frac{\Delta h_{elast}}{\Delta h_{visc}} \approx \frac{E}{\rho_w g R} \approx 10^3, \quad \frac{\Delta h_{elast}}{\Delta d} \approx \frac{EHkh_0}{R^3 \mu d} \left(\frac{B}{\rho_i g H}\right)^n \approx 10^3 - 10^5. \tag{S.43}$$

Thus, the reduction of blister height is dominantly contributed by the elastic relaxation of ice rather than the viscous ice flow.

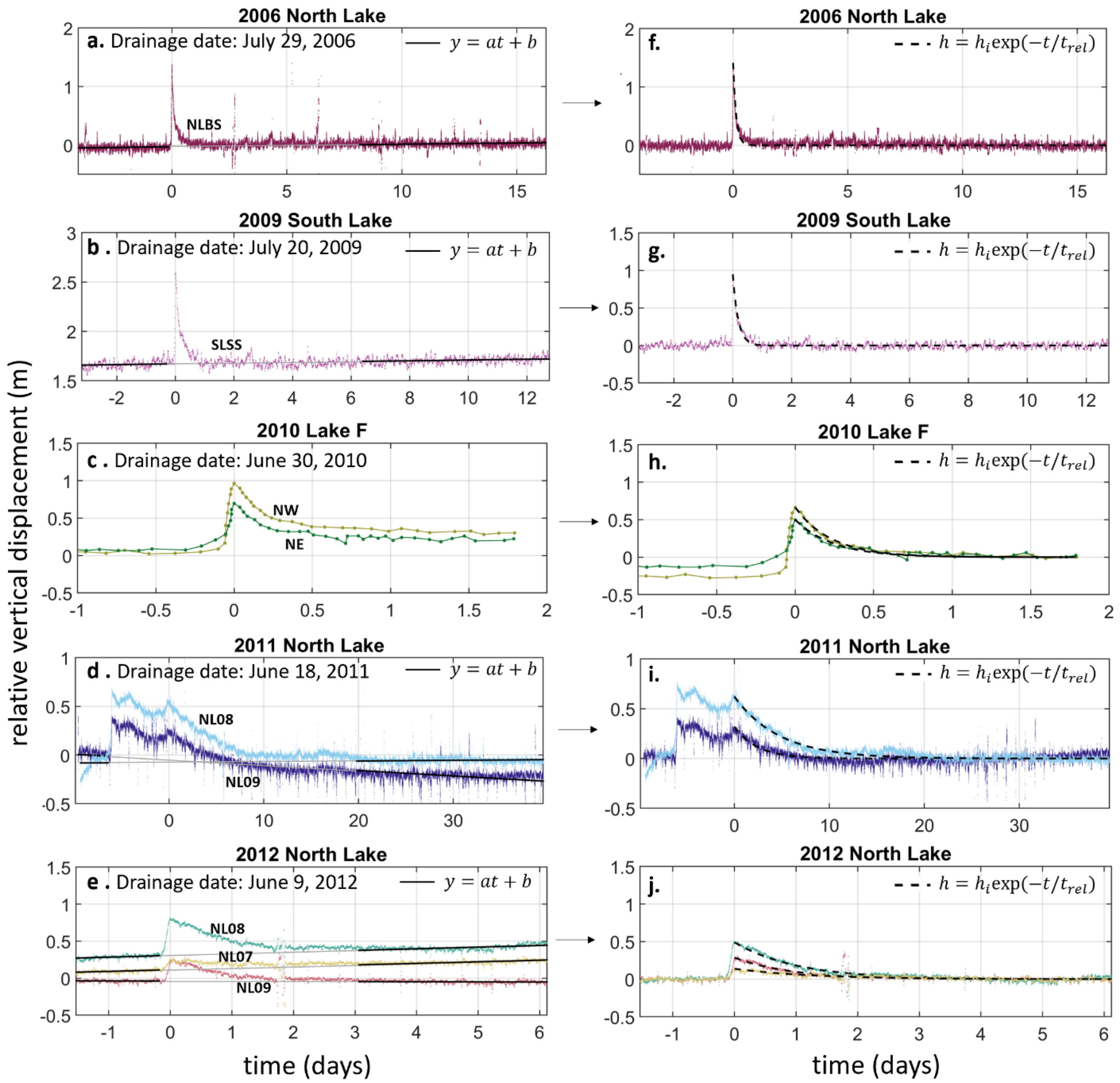

Supplementary Figure 4: GPS uplift data processing. The relative vertical displacement data from individual GPS stations for 5 drainage events **(a-e)** and the detrended data **(f-j)**. The background variation of vertical displacement in time t due to ice-sheet movement is linearly fit to $y = at + b$ (black lines) before and after the uplift peak. The background linear trend is then subtracted from the GPS data to yield the detrended vertical displacements **(f-j)**. Details of the time range used for linear curve fitting are in Methods. The relaxation time t_{rel} (vertical axis in Fig. 3a) is obtained by fitting an exponential curve to the detrended vertical displacements $h(t)$. The 9 detrended vertical displacements are used in Fig. 3b-c.

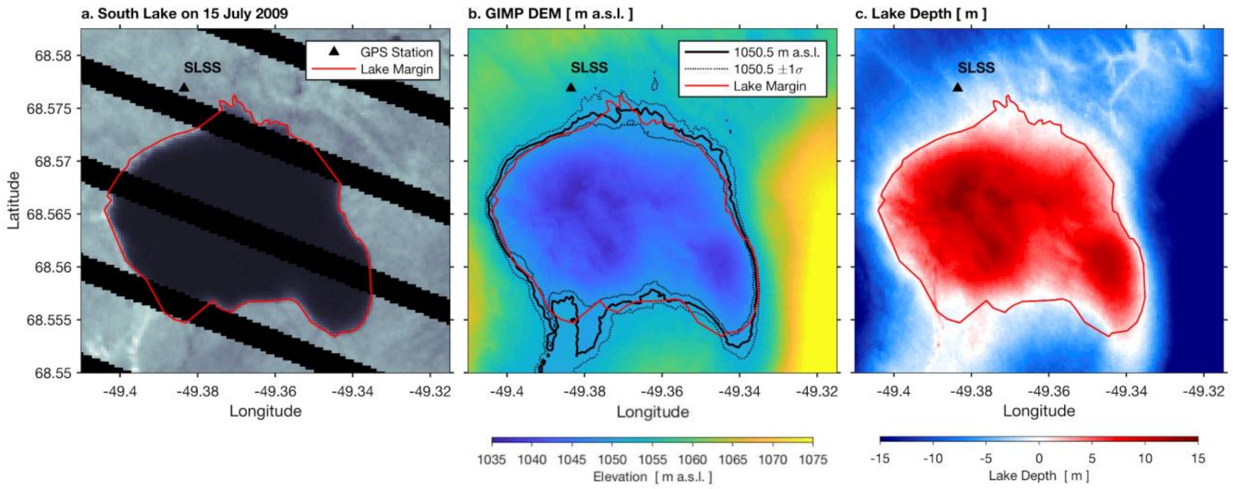

Supplementary Figure 5: South Lake in 2009. **(a)** Landsat 7 image of South Lake on July 15, 2009, five days before rapid drainage. Red line marks lake margin. SLSS GPS station shown with black triangle. **(b)** 30-m resolution MEaSUREs Greenland Ice Mapping Project (GIMP) Digital Elevation Model (DEM) from GeoEye and WorldView Imagery, Version 1 [10,11] for South Lake region. Solid Black contour shows 1050.5 m a.s.l., with dashed contours showing $1050.5 \pm 1\sigma$ = 1050.5 ± 1.27 m a.s.l. **(c)** Lake depth relative to 1050.5 m a.s.l. lake shoreline.

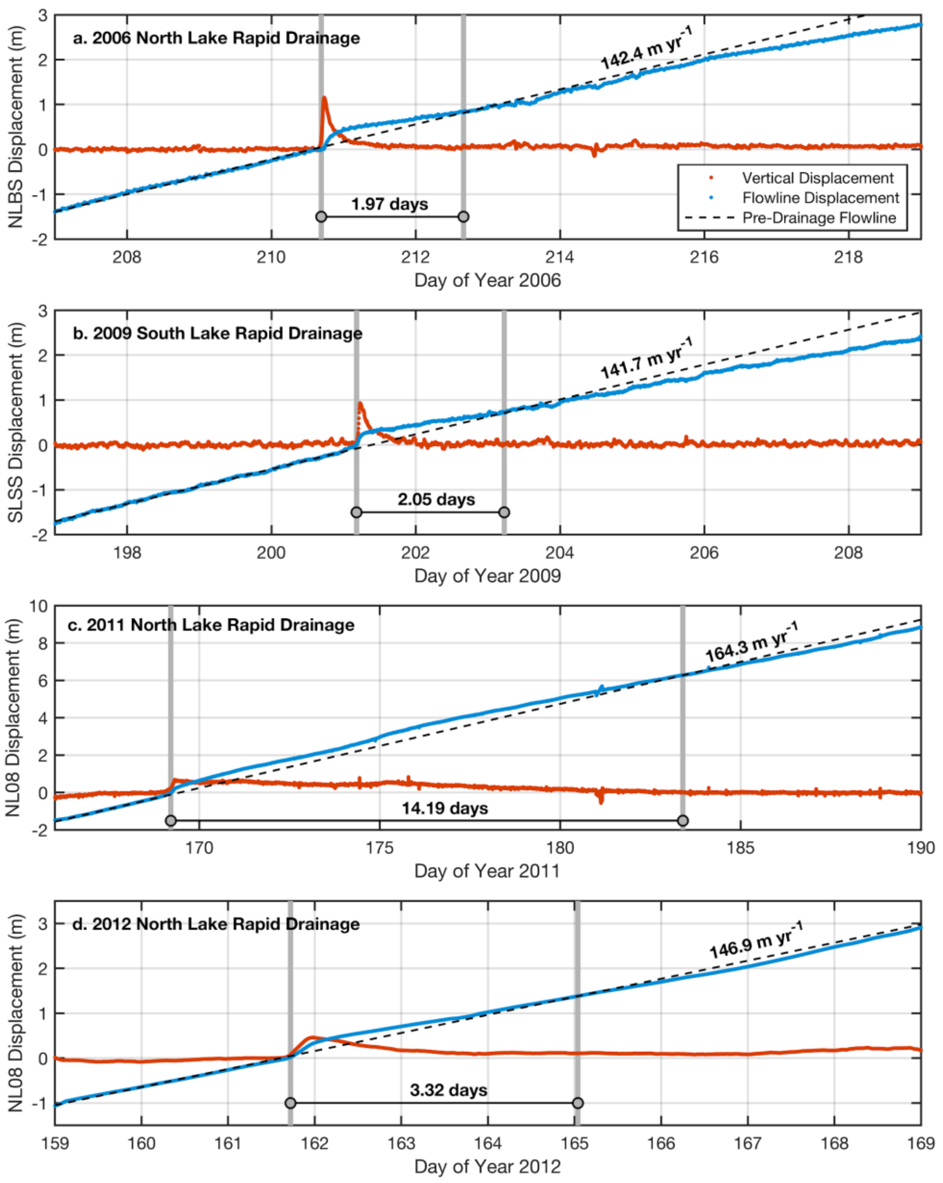

Supplementary Figure 6: Vertical and along-flow displacement during North and South Lake drainages. For all panels, station (red) vertical displacement and (blue) along-flow displacement for an individual GPS station are shown. Dashed black line shows a linear fit to pre-drainage along-flow displacement, with along-flow velocity (slope) noted in the text on panel. Grey vertical bars indicate time of drainage and time of along-flow displacement attaining the value predicted by pre-drainage along-flow velocities, with the duration between these time points in days noted in the text on panel. North Lake drainages for **(a)** 2006, **(c)** 2011, and **(d)** 2012 are shown. South Lake drainage for **(b)** 2009 is shown. The relationship between vertical displacement and along-flow velocity around a moulin has been reported in [12].

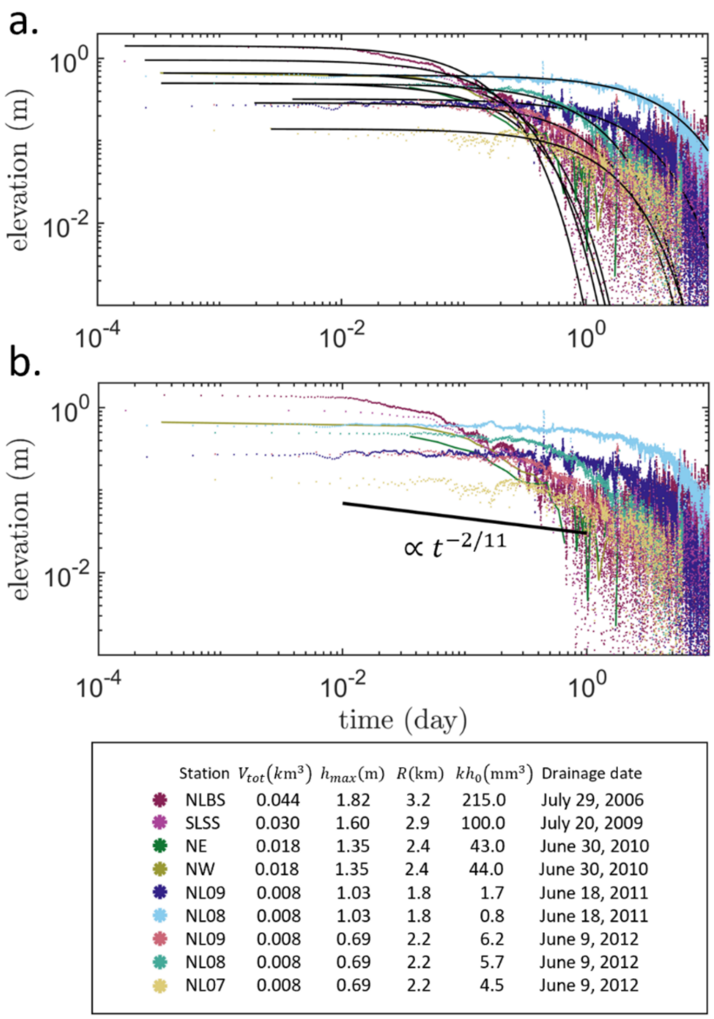

Supplementary Figure 7: Comparison of different models for blister height. **(a)** Our model predicts an exponential decrease of blister height with time (equation (7)). The exponential decay of blister height $h(t) = h_i \exp(-t/t_{rel})$ fitted to all GPS data as a function of time is shown by the solid black curves. The fitted t_{rel} is used to calculate the hydraulic transmissivity (Fig. 3a). The color of each set of uplift data is the same as in Fig. 3. **(b)** Comparison of the GPS data with a power-law (black line) with exponent -2/11 [5].

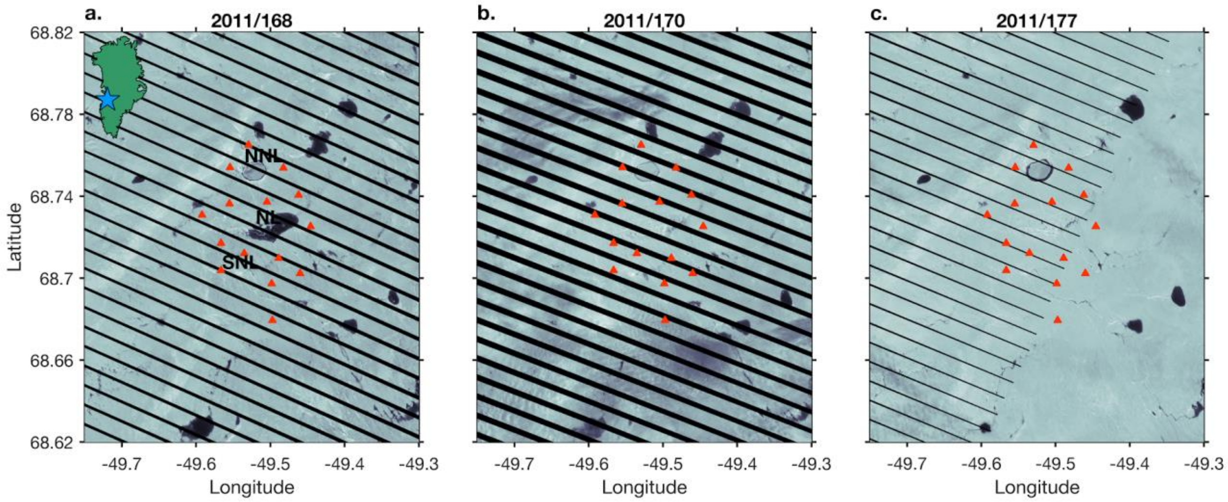

Supplementary Figure 8: Additional surface water injection near North Lake. North Lake (center of a), GPS stations (red triangles), and neighboring lakes in LandSat-7 images bracketing the North Lake drainage event on 2011/169 (day of year 169 in 2011).

Lake name	Lake GPS location	Lake drainage date
North Lake	68.72˚ N, 49.50˚ W	July 29, 2006
		June 18, 2011
		June 9, 2012
South Lake	68.57˚ N, 49.37˚ W	July 20, 2009
Lake F	67.01˚ N, 48.74˚ W	June 30, 2010

Supplementary Table 1: Locations and dates of the lake drainage events.

**Station**	**Year**	$\boldsymbol{V_{tot}}$ **(km)**	$\boldsymbol{h_{max}}$ **(m)**	$\boldsymbol{R}$ **(km)**	$\boldsymbol{kh_0}$ **(mm^3)**
NLBS	2006	±0.010	±0.16	±0.4	±90.2
SLSS	2009	±0.010	±0.13	±0.5	±56.1
NE	2010	±0.027	±0.09	±0.2	±14.4
NW	2010	±0.027	±0.09	±0.2	±15.0
NL09	2011	±0.001	±0.05	±0.1	±0.6
NL08	2011	±0.001	±0.05	±0.1	±0.3
NL09	2012	±0.001	±0.05	±0.2	±2.5
NL08	2012	±0.001	±0.05	±0.2	±2.3
NL07	2012	±0.001	±0.05	±0.2	±1.8

Supplementary Table 2: Parameter uncertainties of field data. Error bars for parameters listed in Fig. 3: lake volume V_{tot}, maximum initial blister height h_{max}, blister radius R, and transmissivity kh_0. The lake volume error bars for the 2009 drainage event are estimated in Methods and for other years are taken from literature [4,13,14]. The errors associated with h_{max}, R, kh_0 are propagated from the lake volume error, as detailed in Methods.

**Parameters**	**Uncertainties**
Elastic Modulus E (Pa)	217 ±30
Permeability k (μm^2)	20 ±2
	52 ±9
	98 ±9
Glycerol Viscosity μ (Pa·s)	0.798 ± 0.005
Pore fraction ϕ	0.50 ± 0.01
Blister radius R (mm)	±1
Fluid in pores radius R_p (mm)	±0.1

Supplementary Table 3: Parameter uncertainties of experimental data. The uncertainties in blister radius and radius of the porous area filled with fluid are due to image processing limitations.

References

[1] C. Y. Lai, Z. Zheng, E. Dressaire, G. Z. Ramon, H. E. Huppert, and H. A. Stone, "Elastic relaxation of fluid-driven cracks and the resulting backflow," *Phys. Rev. Lett*, vol. 117, p. 268001, 2016.

[2] D. A. Spence and P. Sharp, "Self-similar solutions for elastohydrodynamic cavity flow," *Proc. R. Soc. A*, vol. 400, no. 1819, pp. 289–313, 1985.

[3] J. Weertman, "Effect of a basal water layer on the dimensions of ice sheets," *Journal of Glaciology*, vol. 6, no. 44, pp. 191–207, 1966.

[4] L. A. Stevens, M. D. Behn, J. J. McGuire, S. B. Das, I. Joughin, T. Herring, D. E. Shean, and M. A. King, "Greenland supraglacial lake drainages triggered by hydrologically induced basal slip," *Nature*, vol. 522, no. 7554, p. 73, 2015.

[5] D. R. Hewitt, G. P. Chini, and J. A. Neufeld, "The influence of a poroelastic till on rapid subglacial flooding and cavity formation," *J. Fluid Mech.*, vol. 855, pp. 1170–1207, 2018.

[6] I. J. Hewitt, C. Schoof, and M. A. Werder, "Flotation and free surface flow in a model for subglacial drainage. part 2. channel flow," *J. Fluid Mech.*, vol. 702, p. 157, 2012.

[7] M. Morlighem, E. Rignot, J. Mouginot, H. Seroussi, and E. Larour, "Deeply incised submarine glacial valleys beneath the greenland ice sheet," *Nature Geoscience*, vol. 7, no. 6, pp. 418–422, 2014.

[8] K. M. Cuffey and W. S. B. Paterson, *The physics of glaciers.* Academic Press, 2010.

[9] R. LeB. Hooke, "Flow law for polycrystalline ice in glaciers: comparison of theoretical predictions, laboratory data, and field measurements," *Reviews of Geophysics*, vol. 19, no. 4, pp. 664–672, 1981.

[10] I. M. Howat, A. Negrete, and B. E. Smith, "The greenland ice mapping project (gimp) land classification and surface elevation data sets," *The Cryosphere*, vol. 8, no. 4, pp. 1509–1518, 2014.

[11] I. M. Howat, A. Negrete, and B. E. Smith, "Measures greenland ice mapping project (gimp) digital elevation model from geoeye and worldview imagery, version 1," *Boulder, CO: NASA National Snow and Ice Data Center Distributed Active Archive Center*, 2017.

[12] L. C. Andrews, G. A. Catania, M. J. Hoffman, J. D. Gulley, M. P. Lüthi, C. Ryser, R. L. Hawley, and T. A. Neumann, "Direct observations of evolving subglacial drainage beneath the greenland ice sheet," *Nature*, vol. 514, no. 7520, pp. 80–83, 2014.

[13] S. B. Das, I. Joughin, M. D. Behn, I. M. Howat, M. A. King, D. Lizarralde, and M. P. Bhatia, "Fracture propagation to the base of the greenland ice sheet during supraglacial lake drainage," *Science*, vol. 320, no. 5877, pp. 778–781, 2008.

[14] S. H. Doyle, A. L. Hubbard, C. F. Dow, G. A. Jones, A. Fitzpatrick, A. Gusmeroli, B. Kulessa, K. Lindback, R. Pettersson, and J. E. Box, "Ice tectonic deformation during the rapid in situ drainage of a supraglacial lake on the greenland ice sheet," *The Cryosphere*, vol. 7, no. 1, pp. 129–140, 2013.
