# Supplementary material for: Hydraulic transmissivity inferred from ice-sheet relaxation following Greenland supraglacial lake drainages
Source: Nat Commun. 2021 Jun 25;12:3955. doi: 10.1038/s41467-021-24186-6 (PMC8233380; doi:10.1038/s41467-021-24186-6)
Supplement: Supplementary file 4 — Description of additional supplementary files [file 41467_2021_24186_MOESM4_ESM.docx]

Description of additional supplementary information files

Title: Supplementary Movie 1

Description: Top view of the blister experiment shown in Fig. 2.
